# Microbial Composition of Water Kefir Grains and Their Application for the Detoxification of Aflatoxin B1

**DOI:** 10.3390/toxins16020107

**Published:** 2024-02-15

**Authors:** Weidong Ouyang, Zhenlin Liao, Ximiao Yang, Xiao Zhang, Xiaoxuan Zhu, Qingping Zhong, Li Wang, Xiang Fang, Jie Wang

**Affiliations:** Guangdong Provincial Key Laboratory of Nutraceuticals and Functional Foods, College of Food Science, South China Agricultural University, Guangzhou 510642, Chinayxm202220220@163.com (X.Y.); zhangxiaohuge@163.com (X.Z.); zxxdora@163.com (X.Z.); fxiang@scau.edu.cn (X.F.)

**Keywords:** water kefir grains, microbial composition, biosorption, aflatoxin B1, Ames test

## Abstract

Water kefir grains (WKGs), the starter used to develop a traditional beverage named water kefir, consist of a symbiotic mixture of probiotics with diverse bioactivities, but little is known about their abilities to remove mycotoxins that have serious adverse effects on humans and animals. This study investigated the ability of WKGs to remove aflatoxin B1 (AFB1), one of the most toxic mycotoxins, under different settings, and determined the mechanism of absorption mediated by WKGs and the effect of WKGs on the toxicity induced by AFB1 and the reduction in AFB1 in cow milk and tea soups. The results showed the WKGs used herein were dominated by *Lactobacillus*, *Acetobacter*, *Phenylobacterium*, *Sediminibacterium*, *Saccharomyces*, *Issatchenkia*, and *Kodamaea*. HPLC analysis demonstrated that the WKGs effectively removed AFB1 at concentrations ranging from 1 to 5 µg/mL, pH values ranging from 3 to 9, and temperatures ranging from 4 to 45 °C. Additionally, the removal of AFB1 mainly depended on absorption, which was consistent with the Freundlich and pseudo-second-order kinetic models. Moreover, only 49.63% of AFB1 was released from the AFB1-WKG complex after four washes when the release of AFB1 was non-detectable. Furthermore, WKG treatment caused a dramatic reduction in the mutagenicity induced by AFB1 according to an Ames test and reduced more than 54% of AFB1 in cow milk and three tea soups. These results suggested that WKGs can act as a potential bio-absorbent with a high binding ability to detoxify AFB1 in food and feed via a chemical action step and multi-binding sites of AFB1 absorption in a wide range of scenarios.

## 1. Introduction

Mycotoxins, which are toxic secondary metabolites synthesized by filamentous fungi such as *Aspergillus*, *Penicillium*, and *Fusarium*, have contaminated approximately 60–80% of global food and livestock feed, which poses a substantial threat to both human and animal health [1,2,3,4]. As the most toxic contaminant among various mycotoxins, aflatoxin B1 (AFB1), a bifuranocyclic coumarin derivative that is primarily produced by *Aspergillus flavus* and *Aspergillus parasiticus*, exhibits the hepatotoxic, teratogenic, carcinogenic, and immunosuppressive effects in both humans and animals and causes substantial adverse effects on livestock productivity and human health, which causes serious financial losses and threats to public health and safety [5,6]. Therefore, it is urgent to reduce the contamination of AFB1 in food and feed and prevent its toxicity through active strategies, such as physical, chemical, or biological methods.

Compared with physical and chemical strategies, biological treatments exhibit various advantages, such as safety, simplicity, minimal impact on product quality, and cost-effectiveness, and have attracted substantial attention [7,8]. Moreover, the addition of adsorbents to diets contaminated with mycotoxins is widely used to reduce toxin bio-availability in the gastrointestinal tract of animals and detoxify animal feeds [9,10]. Therefore, the use of biomaterials as adsorbents has recently become one of the most promising approaches to removing AFB1 from animal feeds [11,12,13,14,15,16]. Several bacteria and fungi, such as *Lactobacillus* [17,18,19], *Bacillus* [20,21], *Enterococcus* [22], and *Saccharomyces* [23], have been reported to act as bio-adsorbents to remove AFB1. Except for microbial cells, microbial products, such as the cell wall, β-glucan, and humic acid, can also serve as bio-adsorbents for AFB1, as well as aflatoxin M1, a metabolite of AFB1 in the liver when ingested by animals [24,25,26,27,28].

Water kefir, a traditional beverage with a somewhat acidic flavor, an alcoholic fragrance, and a fruity flavor [29,30,31], is typically produced by fermenting a solution of sucrose and fruits with water kefir grains (WKGs), which are non-dairy kefir grains [32]. WKGs are irregular granules that have a cauliflower-like structure, which varies in diameter from 5 to 20 mm, and contain a probiotic consortium that is primarily composed of lactic acid bacteria in the *Lactobacillus* genus, acetic acid bacteria in the *Acetobacter* genus, and yeasts in the *Saccharomyces* genus [32]. The microbial makeup of WKGs depends on their geographical origin and fermentation substrates and conditions [32]. Compared to the single-strain cultures, microbial complexes exhibit significant potential to utilize a variety of substrates and a robust tolerance to adverse environmental circumstances, which results in the widespread use of WKGs in numerous fermented foods, which yields promising outcomes [33]. Recently, studies have demonstrated that water kefir and/or probiotics from WKGs exhibit various health benefits, such as antioxidant and anti-inflammatory properties, antibacterial and antifungal activities, hypocholesterolemic effects, and the capacity to modulate the gut microbiota [32,34,35]. However, little is known about the capacity and mechanism of WKGs with regard to removing hazardous chemicals, such as AFB1, although milk kefir grains, which are dairy kefir grains, and isolated strains of kefir grains can absorb and eliminate AFB1, aflatoxin M1, aflatoxin G1, ochratoxin A, deoxynivalenol, patulin, and zearalenone [36,37,38,39,40]. Therefore, this study was conducted to investigate the characteristics of microbiota in WKGs from Lincang in Yunnan Province of China and evaluate the ability of WKGs to remove AFB1, the effects of fermentation conditions on the removal of AFB1 mediated by WKGs, and the mutagenic potential after the removal of AFB1. Moreover, the potential mechanism of the removal of AFB1 mediated by WKGs was revealed. Furthermore, the effects of the removal of AFB1 mediated by WKGs in cow milk and tea soups were investigated. These findings will help to develop safe and effective bio-adsorbents or biomaterials to detoxify AFB1, which could be used commercially in the food and feed industries, and will help to improve the overall safety and quality of food and feed.

## 2. Results

### 2.1. Microbial Composition of WKGs

**Figure 1:**
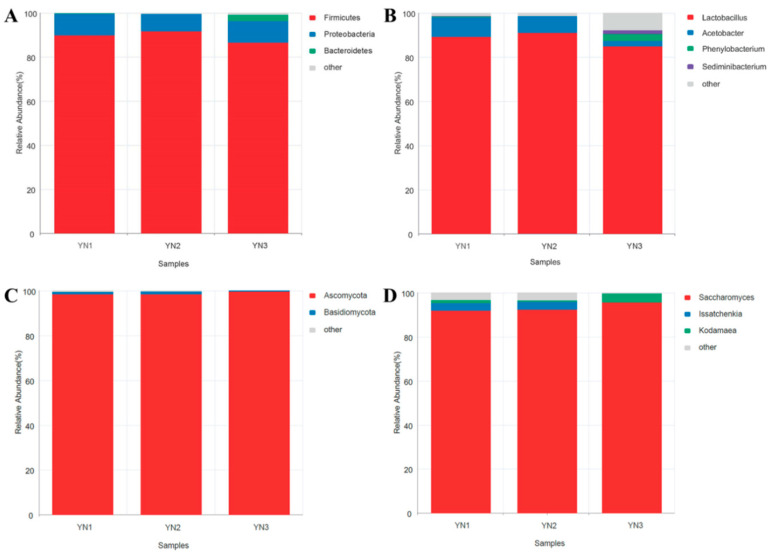
Relative abundance of the bacterial and fungal phyla (**A**,**C**) and genera (**B**,**D**) from the WKGs used to remove AFB1 based on 16s rRNA sequencing (**A**,**B**) and ITS sequencing (**C**,**D**).

A 16S rRNA sequencing analysis revealed that the WKGs used in this study predominantly consisted of bacteria from Firmicutes (89.39%), Proteobacteria (9.18%), and Bacteroidetes (1.06%) (Figure 1A). Additionally, the predominant genera were *Lactobacillus* (88.35%), *Acetobacter* (6.32%), *Phenylobacterium* (1.15%), and *Sediminibacterium* (0.66%) (Figure 1B).

An analysis of ITS sequencing indicated that fungi in the WKGs were primarily composed of Ascomycota (98.96%) and Basidiomycota (0.83%) (Figure 1C). Correspondingly, *Saccharomyces* (93.36%), *Issatchenkia* (2.38%), and *Kodamaea* (1.91%) were found to be predominant in the WKGs (Figure 1D).

### 2.2. Adsorption and Desorption of AFB1 by WKGs

**Figure 2:**
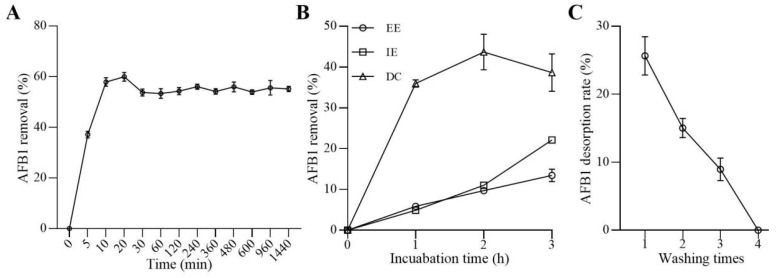
Removal of AFB1 mediated by WKGs when 20% of the WKGs were incubated in brown sugar solution supplemented with 2 µg/mL of AFB1. (**A**) The percentage of AFB1 removed at different times. (**B**) Roles of dead cells (DC), extracellular extracts (EE), and intracellular extracts (IE) of WKGs in the removal of AFB1. (**C**) The rate of AFB1 desorption after washing. Error bars: standard deviation (SD) from three replicates.

When AFB1 was co-incubated with the WKGs, a significant reduction in the content of AFB1 was observed according to a high-performance liquid chromatography (HPLC) analysis. The removal of AFB1 reached 37.12% on average after 5 min and nearly reached the maximum at 20 min when the removal of AFB1 reached 59.96% on average (Figure 2A). Moreover, the removal plateaued out after 30 min when the percentage of AFB1 removed was slightly lower than that at 20 min (Figure 2A). Furthermore, the dead cells of WKGs were the most effective, followed by intracellular extracts and extracellular extracts, which was determined by comparing the percentage of AFB1 removed during a 3 h incubation (Figure 2B). The rates of AFB1 removed for dead cells, intracellular extracts, and extracellular extracts were 35.96%, 4.89%, and 5.86% on average at 1 h; 43.68%, 11.01%, and 9.70% on average at 2 h; and 38.64%, 22.12%, and 13.45% on average at 3 h, respectively. These results demonstrated that the removal of AFB1 caused by WKGs primarily depended on absorption, although degradation also contributed to the removal to some degree, and absorption equilibrium was reached within 30 min.

The releases of AFB1 from the AFB1-WKG complex were evaluated after a series of washes with sterile water. The desorption rates were 25.65%, 15.03%, and 8.95% on average after washing one, two, and three times, respectively (Figure 2C). After the fourth time, almost no detectable AFB1 was released. These results demonstrated that the WKGs retained 50.37% AFB1 after desorption, which suggested that the WKGs could stably bind AFB1.

### 2.3. Effects of the Initial Concentrations of AFB1, Inoculation of WKGs, pH Values, and Temperature on the Removal of AFB1 Mediated by WKGs

**Figure 3:**
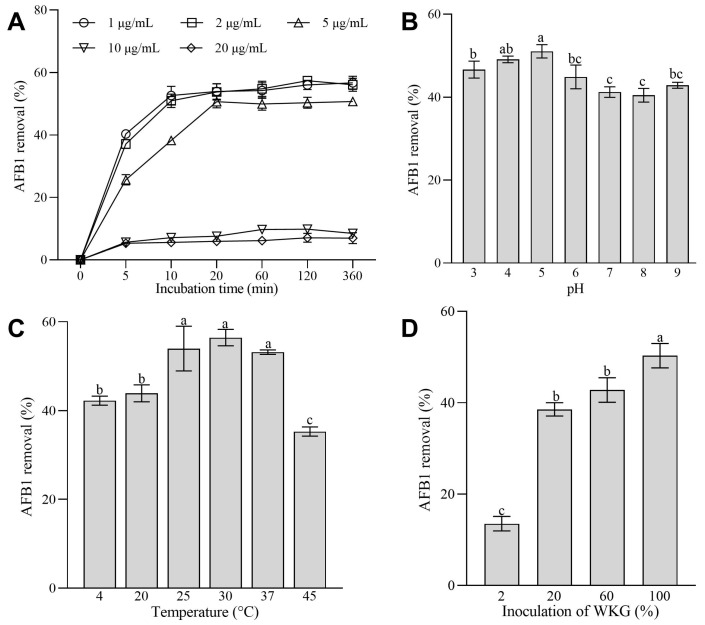
Effects of the initial concentrations of AFB1 (**A**), pH values (**B**), temperature (**C**), and inoculation of WKGs (**D**) on the removal of AFB1 mediated by WKGs. Different lowercase letters on bars denote significant differences (*p* < 0.05). Error bars: SD from three replicates.

In terms of AFB1 concentrations, the WKGs exhibited a comparable ability to remove AFB1 at concentrations that ranged from 1 to 2 µg/mL (*p* > 0.05) (Figure 3A). However, as the AFB1 concentration increased to 5 µg/mL, there was a notable decrease in the percentage of AFB1 removed compared with the 1 and 2 µg/mL AFB1 groups (*p* < 0.05), with a reduction that ranged from 5.72% to 36.44% (Figure 3A). Additionally, the percentages of AFB1 removed were consistently less than 10% in the groups with 10 and 20 µg/mL of AFB1 (Figure 3A).

Moreover, the initial pH values affected the ability of WKGs to remove AFB1. The highest percentage of AFB1 removed (51.05%) was observed at pH 5.0 (Figure 3B). The percentages of AFB1 removed (46.64–51.05%) at low pH values that varied from 3 to 5 were higher than those (40.47–44.88%) at high pH values that ranged from 6 to 9 (Figure 3B), which implies that an acidic environment might aid in the removal of AFB1 mediated by WKGs.

Additionally, the removal of AFB1 increased to 53.97% from 42.24% with an increase in the incubation temperature from 4 to 25 °C (Figure 3C). Moreover, the removal of AFB1 did not change significantly when the temperature varied from 25 to 37 °C (*p* > 0.05), and the maximum percentage of AFB1 removed (56.44%) on average was attained at 30 °C (Figure 3C). In addition, WKGs removed 35.27% of AFB1 at 45 °C, although the estimate was lower by 33.65% than that at 37 °C (Figure 3C).

Furthermore, the inoculation of WKGs significantly affected the removal of AFB1 mediated by WKGs (*p* < 0.05). Compared with that (13.49% on average) in the group of 2% WKGs, the percentage of AFB1 removed in the groups of 20%, 60%, and 100% WKGs increased 1.86-, 2.17-, and 2.73-fold on average, respectively (Figure 3D).

These changes revealed that WKGs could be a bio-adsorbent used in a variety of environments with temperatures that vary from 4 to 45 °C and pH values that vary from 3 to 9 to remove AFB1 at concentrations that vary from 1 to 5 µg/mL.

### 2.4. The Adsorption Isotherms and Kinetics of the Removal of AFB1 by WKGs

The previous finding clearly indicates that adsorption plays a crucial role in the removal of AFB1 mediated by WKGs. To investigate the mechanism of AFB1 absorption, Langmuir and Freundlich adsorption isotherms and kinetics were analyzed. As shown in Table 1, the coefficient of determination (R^2^) in the Freundlich equation was higher than that in the Langmuir, which indicated that the removal of AFB1 mediated by WKGs depended on multi-layer sorption rather than the adsorption of a monolayer on a homogeneous surface.

Moreover, the adsorption kinetics were analyzed using a pseudo-first/second-order kinetic model and an intraparticle diffusion model to investigate the adsorption efficiency. The values of R^2^ of the first-order and second-order kinetic model and the intraparticle diffusion model were 0.710, 0.9995, and 0.663, respectively (Table 2), and the calculated adsorption capacity (Qeq, cal) estimate (6.878) was close to the experimental (Qeq, exp) value (6.896) under the pseudo-second-order kinetic model. These findings demonstrated that the adsorption of AFB1 mediated by WKGs fitted to the pseudo-second-order model, which indicated that the rate of adsorption in the removal of AFB1 mediated by WKGs is determined by the chemical action step rather than by diffusion [41]. 

### 2.5. Analysis of AFB1 and AFB1 Metabolites by HPLC–Quadrupole Time-of-Flight Mass Spectrometry (HPLC-Q-TOF-MS)

An HPLC-Q-TOF-MS analysis showed that the content of AFB1 decreased significantly when 20% of WKGs were added into the brown sugar solution containing 2 µg/mL of AFB1 and co-incubated at 25 °C for 30 min (Figure S1). Moreover, compared with AFB1 and the metabolites produced by WKGs in the brown sugar solution, nine new peaks were observed in the co-incubation cultures of AFB1 and WKGs (Figure S2). Their molecular formulae are C_10_H_2_O_12_ (*m*/*z* 313.97), C_6_H_12_O_5_ (*m*/*z* 164.08), C_10_H_16_O_3_ (*m*/*z* 184.11), C_21_H_12_O_3_ (*m*/*z* 312.09), C_9_H_6_O_3_ (*m*/*z* 162.04), C_10_H_21_O_3_ (*m*/*z* 237.14), C_15_H_6_O_3_ (*m*/*z* 234.04), C_15_H_22_O_2_ (*m*/*z* 234.17), and C_20_H_22_O_5_ (*m*/*z* 342.15) based on an analysis with MassHunter Qualitative Analysis B.08.00 (Agilent Technologies, Santa Clara, CA, USA).

### 2.6. The Ames Test for Mutagenicity

**Figure 4:**
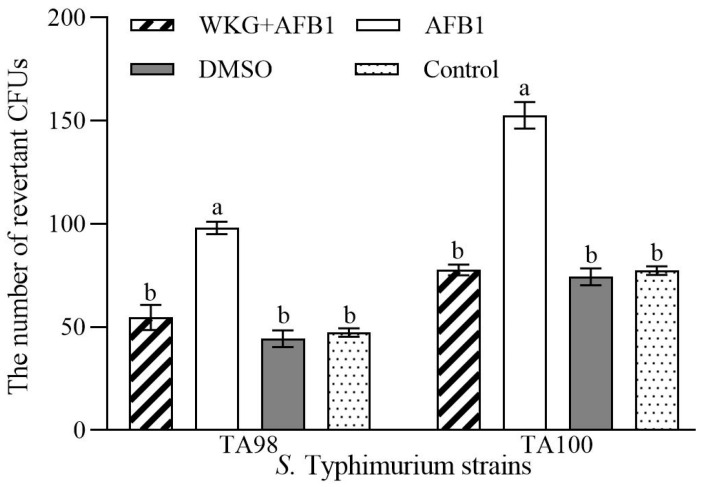
Reduction caused by the WKGs in the mutagenicity induced by AFB1. The number of revertant CFUs of *S.* Typhimurium TA98 and TA100 among the different groups. AFB1 group indicates extracts from brown sugar solutions containing 5 µg AFB1. The group of WKG + AFB1 indicates extracts from the 30 min co-incubation of 5 µg AFB1 and WKGs in brown sugar solutions. The control and DMSO groups mean extracts from the sterilized brown sugar solution and DMSO solution, respectively. Different lowercase letters on bars denote significant differences (*p* < 0.05). Error bars: SD from three replicates.

To assess the toxicity following the removal of AFB1 mediated by WKGs, mutagenicity was measured using the Ames test. Compared to the AFB1 group, the number of revertant colony-forming units (CFUs) in the samples after the removal of AFB1 significantly decreased by 44.89% for *S.* Typhimurium TA98 and 49.13% for TA100 (*p* < 0.05) (Figure 4). The estimates in the sample after the removal of AFB1 were similar to those observed in the control and DMSO groups (*p* > 0.05). These findings demonstrated that WKGs could lower the amount of AFB1 in the samples, and then, decrease or even eliminate the mutagenicity caused by AFB1.

### 2.7. Removal of AFB1 by WKGs in Cow Milk and Teas Contaminated with AFB1

**Figure 5:**
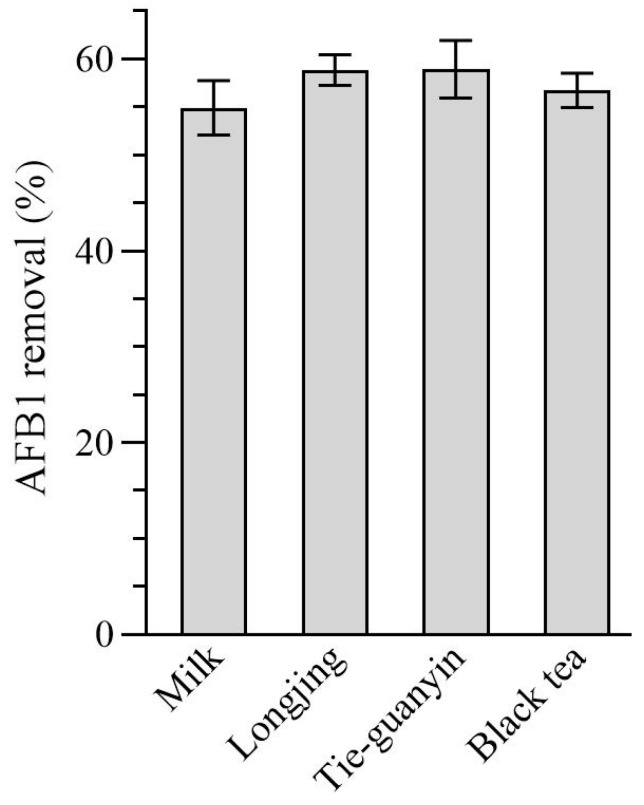
The removal of AFB1 mediated by WKGs in cow milk and tea soups contaminated with AFB1. Volumes of 5 mL of cow milk or tea soups derived from water immersion solution of Longjing tea, Tie-guanyin tea, or Chinese black tea were added directly to 2 µg/mL of AFB1 to prepare cow milk or tea soups contaminated with AFB1. Volumes of 5 mL of AFB1 contaminated cow milk and tea soups were incubated with 1 g of activated WKGs at 25 °C for 30 min with shaking (180 rpm). Error bars: SD from three replicates.

It has been reported that AFB1 can be detected in milk, dairy products, and teas [42,43,44,45]. Thus, tea soups produced using three teas and cow milk contaminated with AFB1 were chosen to assess the efficacy of WKGs in the removal of AFB1 in various food matrices. When co-incubated in cow milk, Longjing tea (non-fermented tea) soup, Tieguan-yin tea (semi-fermented tea) soup, or black tea (fermented tea) soup contaminated with 2 µg/mL of AFB1, WKGs decreased the content of AFB1 by 54.90%, 58.85%, 58.96%, and 56.75% on average (Figure 5), respectively. This finding demonstrated the potential of WKGs for the effective reduction in AFB1 in various food matrices.

## 3. Discussion

Kefir grains contain symbiotic probiotic consortia that primarily consist of lactic acid bacteria, acetic acid bacteria, and/or yeast. The microbial makeup of the kefir grains depends on the types of kefir grains, the geographical origin, and the circumstances and substrates used during fermentation [34]. Based on the substrate that is used, kefir grains are divided into dairy kefir grains (i.e., milk kefir grains) and non-dairy kefir grains (i.e., water kefir grains), which differ based on the characteristics of the grains, the types of grain exopolysaccharide, the microbial community, and the diversity of consumers [32]. It was reported that milk kefir grains and their microorganisms, such as *Kazachstania unisporus*, *Lactobacillus*, and *Saccharomyces cerevisiae*, could remove mycotoxins, such as AFB1, aflatoxin M1, aflatoxin G1, ochratoxin A, and zearalenone [37,38]. However, the effects of WKGs on the removal of mycotoxins are little known, although water kefir and/or its extracts exhibit various bioactivities, such as antioxidant, antimicrobial, and anti-inflammatory effects [32]. In this study, WKGs sourced from Lincang in Yunnan Province of China were found to detoxify AFB1 through multi-layer sorption via chemical action and degradation, as discussed below.

It was reported that WKGs can contain a variety of bacteria, including lactic acid bacteria, such as *Lactobacillus*, *Lactococcus*, *Leuconostoc*, *Bifidobacteria*, *Pediococcus*, and *Oenococcus*; acetic acid bacteria, such as *Acetobacter*, *Gluconobacter*, and *Gluconacetobacter*; and other bacteria, such as *Zymomonas*. They can also contain yeast, such as *Saccharomyces*, *Kluyveromyces*, *Dekkera*, *Hanseniaspora*, *Kazachstania*, *Torulaspora*, *Pichia*, *Zygotorulaspora*, and *Yarrowia* [32]. In this study, the WKGs were primarily composed of bacteria that were members of *Lactobacillus*, *Acetobacter*, *Phenylobacterium*, and *Sediminibacterium* and fungi that were members of *Saccharomyces*, *Issatchenkia*, and *Kodamaea*. Previous studies have indicated that *Lactobacillus* spp., *Acetobacter* spp., and *Saccharomyces cerevisiae* can remove AFB1 through absorption [39,46,47]. Thus, it was hypothesized that *Lactobacillus*, *Acetobacter*, and *Saccharomyces* might be the predominant microorganisms in WKGs during the removal of AFB1.

Additionally, 20% of WKGs removed 59.96% of AFB1 at the concentration of 2 µg/mL within 20 min (i.e., the capacity of WKGs for the adsorption of AFB1 is 29.98 µg/g), which is more effective than that reported in 20% of milk kefir grains with an AFB1 adsorption capacity of 0.48 µg/g [36]. Additionally, the removal of AFB1 mediated by WKGs was associated with both absorption and degradation, and absorption played dominant roles and fitted the Freundlich isotherm and pseudo-second-order model, which was similar to the findings reported in the removal of ochratoxin A mediated by Tibetan kefir grains [33]. This suggested that the rate of adsorption of AFB1 mediated by WKGs depended on the chemical action step and multi-binding sites of AFB1 absorption [48]. In addition, the adsorption of mycotoxins by bacteria and fungi is reversible, and mycotoxins might be released from microbial cells when they are only absorbed with weak non-covalent binding, which could lead to potential harm to the health of host [19]. In this study, the WKGs retained 74.32% AFB1 after one wash and 50.37% AFB1 after four washes when no AFB1 could be released from the AFB1-WKGs complex. The estimates were close to this, i.e., 62% AFB1, in the AFB1-KG complex after the sequential desorption of AFB1 from the milk kefir grains [39], which suggested that WKGs had high binding potential, which prevented the release of AFB1 from the AFB1-WKG complex into humans or animals. In addition to absorption, degradation was another way for WKGs to remove AFB1. When AFB1 was co-incubated with WKGs, nine unknown metabolites were observed, suggesting that WKGs could degrade some of AFB1 into unknown metabolites or WKGs might produce new metabolites with the help of AFB1. The observation of unknown metabolites is similar to the findings of previous reports [49,50,51].

Moreover, the initial concentrations of AFB1, pH values, temperature, and inoculation of WKGs affected the ability of WKGs to remove AFB1. The removal of AFB1 decreased as the concentration of AFB1 increased from 2 to 20 µg/mL, which was similar to the removal of zearalenone by *Lactobacillus* strains [52]. Acidic conditions with pH values that varied from 3 to 5 could facilitate the removal of AFB1 mediated by WKGs, which was similar to previous studies on the effects of pH values on the removal of ochratoxin A mediated by Tibetan kefir grains [33]. The amount of AFB1 removed decreased as the pH values increased from 5 to 9, which was similar to the studies on the effects of pH on the removal of AFB1 mediated by *Lactobacillus plantarum* [53]. The increase in pH facilitates cation exchange, which then results in an increase in desorption and a decrease in adsorption [54,55,56], which might account for the higher percentage of AFB1 removed mediated by WKGs at pH values that varied from 3 to 5 compared with that at pH values that varied from 6 to 9. Moreover, the reduction in AFB1 increased with an increase in the temperature in the range of 4 to 25 °C, and the reduction in AFB1 did not differ significantly between 25 and 37 °C. However, it was lowered at 45 °C when the WKGs were co-incubated with AFB1. The changes caused by different temperatures in the removal of AFB1 mediated by WKGs were similar to the results documented on the reduction in ochratoxin A mediated by Tibetan kefir grains [33] and indicated that the WKGs used in this study could be applied in environments with a relatively wide range of temperatures, as well as both in vitro and in vivo. High temperature may have an impact on the bacterial surface, which leads to the formation of extra bonds with AFB1 [57]. This could induce the denaturation of the AFB1-binding protein, which would then facilitate the binding of AFB1 to the protein structure [58]. This could explain the increases in the removal of AFB1 as the temperature increased from 4 to 25 °C and decreased during the removal of AFB1 at 45 °C. In addition, the average percentage of AFB1 removed by WKGs increased with the increase in the inoculation of WKGs, which was similar to the increase in the removal of ochratoxin A when the inoculation of Tibetan kefir grains increased from 2 to 3% [33]. 

Furthermore, accompanied by the reduction in AFB1, the mutagenicity of the samples treated with WKGs was lost, which was similar to the previous findings, including the dietary intervention of *L. plantarum*, which promotes the adsorption of AFB1, against the toxicity of AFB1 [19], and the protective effects of dietary Kefir against the hepatotoxicity induced by AFB1 in Nile tilapia fish (*Oreochromus niloticus*) [59]. 

Finally, WKGs could remove 54.90% of AFB1 from cow milk, which is similar to the detoxification of AFB1 by *L. plantarum* and prebiotics in cow milk [43]. Moreover, tea, a particularly popular economic crop worldwide, could be contaminated by AFB1 during planting, harvesting, processing, and storing, which causes serious problems for the quality and safety of the tea industry [60]. It was first reported that kefir grains could reduce more than 56% of AFB1 in three different tea soups. 

Overall, these findings suggest that WKGs can be used as a promising bio-carrier to effectively and safely reduce AFB1 contamination and limit its toxicity in food and feed. 

## 4. Conclusions

The WKGs were composed of *Lactobacillus*, *Acetobacter*, *Phenylobacterium*, *Sediminibacterium*, *Saccharomyces*, *Issatchenkia*, *Kodamaea*, and others, and could effectively detoxify AFB1 at concentrations that ranged from 1 to 5 µg/mL at pH values that varied from 3 to 9 and temperatures that ranged from 4 to 45 °C. Moreover, the removal of AFB1 was primarily attributed to multi-binding site absorption through the chemical action step of cells, which depended on the inoculation of WKGs. In addition to absorption, WKGs also degraded some of AFB1 into other metabolites. Furthermore, WKGs could significantly reduce the mutagenicity induced by AFB1 and effectively remove AFB1 in foods, such as cow milk and tea soups. To our knowledge, this is the first study to reveal that WKGs can act as a potential bio-absorbent or biocarrier in the removal and detoxification of AFB1 from liquid matrices, paving the way for potential commercial use in the production of food and feed to remove and detoxify AFB1. However, further studies are needed to unveil the crucial strains of WKGs for this purpose and the structures of the degradation products, and illuminate the protection of WKGs against toxicities induced by AFB1 in vivo.

## 5. Materials and Methods

### 5.1. Activation of WKGs

A total of 15 g of brown sugar was dissolved in 150 mL of hot water and boiled in a water bath for 20 min. After the solution had cooled to room temperature, WKGs (9 g) derived from Lincang in Yunnan Province of China were supplemented and incubated at 25 °C without shaking. After 48 h, bubbles and a sweet–sour aroma appeared and the solution was filtered with sieving to obtain fresh WKGs. After activation twice, the activated WKGs were rinsed with deionized water and used in the following experiments.

### 5.2. Analysis of Microbial Community Composition

The total DNA from the WKGs was extracted using an EZNA™ Mag-Bind Soil DNA Kit (Omega BioTek, Norcross, GA, USA). The concentration and quality of the extracted DNA were evaluated using a Qubit 4.0 (Thermo Fisher Scientific, Waltham, MA, USA). Subsequently, the 16S rRNA V3-V4 amplicon was amplified using the primers F (5′-CCTACGGGNGGCWGCAG-3′) and R (5′-GACTACHVGGGTATCTAATCC-3′) and the ITS sequence was amplified using the primers F (5′-CTTGGTCATTTAGAGGAAGTAA-3′) and R (5′-GCTGCGTTCTTCATCGATGC-3′) under the action of a 2×Hieff^®^ Robust PCR Master Mix (Yeasen, Shanghai, China). After PCR amplification, the resulting products were validated through electrophoresis in 2% (*w*/*v*) agarose gels and were purified and quantified using a Qubit^®^ 4.0 Green double-stranded DNA assay and a bioanalyzer (Agilent 2100, Agilen Technologies, Santa Clara, CA, USA), respectively. Then, the purified PCR amplicons were sequenced using an Illumina MiSeq platform (Illumina, Inc., San Diego, CA, USA). The raw Illumina sequences were deposited in the NCBI Sequence Read Archive under the project accession number PRJNA991295. Quality filtering of the raw sequences was conducted using Trimmomatic (version 3.3, http://www.usadellab.org/cms/?page=trimmomatic, accessed on 23 October 2023). Operational taxonomic units (OTUs) sharing a 97% similarity threshold were analyzed using Usearch software (version 11.0.667, https://www.drive5.com/usearch/, accessed on 23 October 2023) and subsequently taxonomically classified utilizing the RDP Database and UNITE fungal ITS Database.

### 5.3. Removal of AFB1 by WKGs

A total of 1 g of activated WKGs was incubated in a 5 mL solution of sterilized brown sugar that contained 2 µg/mL of AFB1 (J&K Scientific, Beijing, China) for 24 h at 25 °C with shaking at 180 rpm. During the 24 h incubation, the brown sugar solution that contained AFB1 served as the control group. According to previous studies, half the volume of chloroform was used to extract the supernatant three times via shaking at 180 rpm [61,62,63]. The resulting chloroform fractions were subsequently evaporated and dissolved in dimethyl sulfoxide (DMSO, Sigma-Aldrich, St. Louis, MO, USA). After filtration through a 0.22 µm pore filter, the redissolved solution was stored at −20 °C for subsequent HPLC and HPLC-Q-TOF-MS analyses. Approximately 94–96% of AFB1 could be recovered from the brown sugar solution by chloroform extraction. All the experiments were repeated three times.

### 5.4. Quantification of AFB1 Using HPLC

The AFB1 analysis was conducted using HPLC (Waters Alliance 2695; Milford, MA, USA) equipped with a YMC C18 column (250 × 4.6 mm, 5 µm) and a UV detector [64]. The injection volume of the samples was set at 10 µL, and the mobile solvent was composed of a mixture of water–methanol–acetonitrile at 48:40:12 (*v*/*v*/*v*). The elution was conducted at a constant flow rate of 0.8 mL/min. The column temperature and wavelength of the UV detector were set at 30 °C and 360 nm, respectively. The limit of detection for AFB1 was determined to be 0.01 µg/mL, and the limit of quantitation was established at 0.05 µg/mL under the given experimental conditions used. Standard solutions containing 1, 2, 5, 10, 20, or 50 μg/mL of AFB1 were used as external standards to ensure the quality of the HPLC data. Moreover, the external calibration curves of the standard AFB1 solutions were used to determine the AFB1 concentrations in the samples. To ascertain the efficacy of the removal of AFB1, the following formula was utilized: 

AFB1 removal (%) = (1 − remaining AFB1 in sample)/(total AFB1 in control sample) × 100.

### 5.5. Removal of AFB1 by Dead Cells, and Extracellular and Intracellular Extracts

The solution of activated WKGs (20%) was centrifuged at 12,000 rpm for 15 min at 4 °C to collect the supernatant. The obtained supernatant was filtered using a 0.22 µm pore filter to yield the extracellular extracts for the removal of AFB1, and the resulting WKGs were washed twice with 10 mM phosphate buffer (pH 8.0), resuspended in an equivalent volume of 10 mM phosphate buffer (pH 8.0), and subjected to ultrasonication (25 kHz, ultrasound for 4 s intervals of 1 s, 20 min) in an ice bath. The resulting ultrasonicated mixture was centrifuged at 12,000 rpm at 4 °C. After 15 min, the supernatant was collected and filtered using a 0.22 µm pore filter, which yielded intracellular extracts for the removal of AFB1. To yield dead cells, an equal amount of activated WKGs was washed with 10 mM phosphate buffer (pH 8.0), boiled for 20 min, and resuspended in an equal volume of 10 mM phosphate buffer (pH 8.0). The dead cells, extracellular extracts, and intracellular extracts were incubated with 2 µg/mL of AFB1 at 25 °C while agitating at 180 rpm for 3 h. A brown sugar solution containing 2 µg/mL of AFB1 served as the control for the extracellular extracts, and phosphate buffer containing 2 µg/mL of AFB1 served as the control for the intracellular extracts and dead cells. In the control group, all the other variables were identical to those for the corresponding experimental groups. At 5 min intervals, the HPLC analysis described above was used to quantify the residual AFB1. All the experiments were repeated three times.

### 5.6. AFB1 Release from WKGs

The release of AFB1 was measured as previously described [65] with slight modifications. Briefly, 1 g of the activated WKGs was incubated in 5 mL of brown sugar solution that contained 2 µg/mL of AFB1 at 25 °C. After shaking for 30 min at 180 rpm, the solution was centrifuged at 12,000 rpm at 4 °C for 15 min. The AFB1 concentration was quantified in the resulting supernatant by HPLC as described above, and the total AFB1 absorbed in the WKGs was calculated using the following formula: 

Total AFB1 absorbed in WKGs = (2 − AFB1 concentration in the supernatant) × 5. 

The precipitates were simultaneously resuspended in 5 mL of fresh sterilized water and incubated at 25 °C with shaking at 220 rpm. After 20 min, the solution was centrifuged and the AFB1 concentration was measured in the supernatant by HPLC, while the precipitates were collected. The amount of AFB1 released from the WKGs was calculated using the following formula: 

The amount of the released AFB1 from WKGs = AFB1 concentration in the supernatant × 5 

After the experiments described above had been repeated four times, the desorption Rate (R) was calculated using the following formula: 

R (%) = (1 − released AFB1 from WKGs/total AFB1 absorbed in WKGs) × 100. 

All the experiments were repeated three times.

### 5.7. Effect of the Initial Concentration of AFB1, Inoculation of WKGs, pH, and Temperature on the Removal of AFB1

To investigate the effects of the concentration of AFB1 on its removal, initial concentrations of AFB1 in the brown sugar solution were established at 1, 2, 5, 10, or 20 µg/mL. A volume of 5 mL of the brown sugar solution supplemented with AFB1 was co-incubated with 1 g of WKGs at 25 °C for 360 min with shaking at 180 rpm. The brown sugar solution supplemented with 1, 2, 5, 10, or 20 µg/mL of AFB1 served as the control.

To investigate the effects of initial pH on the removal of AFB1, the initial pH values in the brown sugar solution supplemented with 2 µg/mL of AFB1 were established at 3.0, 4.0, 5.0, 6.0, 7.0, 8.0, or 9.0. Then, 5 mL of the solution was added to 1 g of the WKGs at 25 °C for 30 min with shaking at 180 rpm. A volume of 5 mL of brown sugar solution at pH 3.0, 4.0, 5.0, 6.0, 7.0, 8.0, or 9.0 that contained 2 µg/mL of AFB1 served as the control. 

To evaluate the effect of temperature on the removal of AFB1, a volume of 5 mL of brown sugar solution that contained 2 µg/mL of AFB1 was co-incubated with 1 g of WKGs at 4, 20, 25, 30, 37, or 45 °C for 30 min with shaking at 180 rpm. In the control group, a volume of 5 mL of brown sugar solution that contained 2 µg/mL of AFB1 was incubated at 4, 20, 25, 30, 37, or 45 °C for 30 min with shaking at 180 rpm. 

The effect of WKG inoculation was determined by inoculating 2, 20, 60, or 100% (*w*/*v*) of WKGs. A volume of 5 mL of brown sugar solution that contained 2 µg/mL of AFB1 was co-incubated with WKGs at 25 °C for 30 min with shaking at 180 rpm. The brown sugar solution that contained 2 µg/mL of AFB1 served as the control. 

After the incubation, the HPLC analysis described above was used to quantify the residual AFB1. All the experiments were repeated three times.

### 5.8. Adsorption Isotherms and Kinetics

A total of 1 g of WKGs was incubated with 5 mL of brown sugar solution supplemented with AFB1 at different concentrations that varied from 2 to 20 µg/mL for 120 min at 25 °C with shaking. The culture was sampled following incubation at 5, 10, 20, 30, 60, or 120 min and centrifuged at 12,000 rpm for 15 min. The concentration of AFB1 in the resulting supernatant was quantified using HPLC. The absorption isothermal curves of AFB1 are depicted, and the adsorption isotherms were calculated according to the Langmuir (Equation (1)) and Freundlich (Equation (2)) adsorption models.

1/q_e_ = 1/q_m_ + (1/(q_m_ × K_L_)) × 1/C_e_ (1)

ln q_e_ = ln K_f_ + (1/n) × ln C_e_ (2)

In the above equations, C_e_ and q_e_ are the concentration of AFB1 in the solution (µg/mL) and the adsorption capacity at equilibrium, respectively; q_m_ is the maximum adsorption capacity of AFB1 (µg/g); K_L_ is the Langmuir constant associated with free energy and the affinity of the binding sites (mL/µg); and K_f_ and n are the Freundlich constants. 

Additionally, the adsorption kinetics were assayed using kinetic models [40], including the pseudo-first-order (Equation (3)) and pseudo-second-order (Equation (4)) kinetic models and the intraparticle diffusion model (Equation (5)).

lg (q_e_ − q_t_) = lg q_e_ − (k_1_/2.303) × t (3)

t/q_t_ = 1/(k_2_ × (q_e_)^2^) + (1/q_e_) × t (4)

q_t_ = k_3_ × t^1/2^ + b (5)

In Equation (3), q_e_ and q_t_ are the amounts of AFB1 adsorbed on the WKGs (µg/g) at equilibrium and at different times t (min), and k_1_ (min^−1^) is the rate constant for the adsorption process. q_e_ and k_1_ were calculated from the intercept and the slope of the pseudo-first-order straight line.

In Equation (4), k_2_ (g/mg min) is the rate constant, and the values of q_e_ and k_2_ were derived from the intercept and slope of a linear plot of t/q_t_ versus t.

In Equation (5), b is the intercept whose values represent the boundary layer thickness associated with the boundary layer effect. k_3_ is the intraparticle diffusion constant (mg/g min^1/2^). The values of k_3_ and b were obtained from the slope and intercept of a linear plot of q_t_ versus t^1/2^.

### 5.9. Analysis of AFB1 and AFB1 Metabolites by HPLC-Q-TOF-MS

After 30 min co-incubation of the WKGs with 2 µg/mL of AFB1 at 25 °C with shaking, chloroform was used to extract the supernatant as described above. The resulting chloroform fractions were separated using an Agilent 1290 system (Agilent Technologies, Santa Clara, CA, USA) equipped with an auto-injector and quaternary HPLC pump (Agilent Technologies, Santa Clara, CA, USA) [61,62,63]. The chromatographic separations were conducted using an Eclipse Plus C18 column (100 × 2.1 mm, 1.8 μm,) (Agilent Technologies, Santa Clara, CA, USA) with a mobile solvent that consisted of a mixture of methanol–water at 45:55 (*v*/*v*), as well as 0.1% formic acid. The injection volume was 10 µL and the total run time was 15 min with a flow rate of 0.3 mL/min. An MS analysis was performed with a 6540 UHD ESI Q-TOF instrument (Agilent Technologies, Santa Clara, CA, USA) in the positive ionization mode and full scan mode. The parameters were as follows: the capillary voltage, dry temperature, flow rate of the drying gas, and nebulizer were set at 4.5 kV, 180 °C, 5.0 L/min, and 0.8 bar, respectively. The data were collected within the range of *m*/*z* 100–2000. An MS analysis of the chemical compounds was performed using MassHunter Qualitative Analysis B.08.00 (Agilent Technologies, Santa Clara, CA, USA). After 30 min incubation at 25 °C with shaking, samples extracted from the brown sugar solutions with 2 µg/mL of AFB1 were used as the positive control, and samples extracted from the brown sugar solutions and cultures of WKGs in brown sugar were used as the negative control. The standard solutions containing 1, 2, 5, 10, 20, or 50 μg/mL of AFB1 were used as external standards to ensure the quality of the HPLC-Q-TOF-MS data. All the experiments were repeated three times.

### 5.10. Ames Mutagenicity Assay

To assess the mutagenicity of the samples after the removal of AFB1, an S9 Enzyme Activation kit (Iphase Pharma Service, Beijing, China) was used to conduct a Salmonella (Ames) test as described previously [6,66]. Briefly, the products obtained from 30 min co-incubation with WKGs and AFB1 were incubated with *Salmonella* Typhimurium TA98 and TA100 strains for 48 h at 37 °C. The number of colonies of *S.* Typhimurium was quantified and expressed as the number of revertant CFUs. The positive controls consisted of samples derived from brown sugar solutions containing AFB1, while the samples derived from the brown sugar solution and DMSO served as the negative control in these experimental setups. All the experiments were repeated three times.

### 5.11. Removal of AFB1 in Milk and Tea

To explore the possible use of WKGs as absorbents to remove AFB1 in food, activated WKGs were incubated with cow milk and tea soups that were contaminated with AFB1. Briefly, 0.5 g of Longjing tea (Hangzhou West Lake Longjing Tea Co., Ltd., Hangzhou, China), Tie-guanyin tea (BAMA TEA Co., Ltd., Fujian, China), or Chinese black tea (Wuhu chaye Co., Ltd., Fujian, China) was immersed in 10 mL of warm water at 60 °C for 15 min, and the supernatant was collected through centrifugation to yield the tea soups. A total of 5 mL of cow milk (Inner Mongolia Yili Industrial Group Co., Ltd., Hohhot, China) or the tea soups was directly added to 2 µg/mL AFB1 to prepare the cow milk or tea soups contaminated with AFB1. A total of 5 mL of cow milk and the tea soups contaminated with AFB1 was incubated with 1 g of the activated WKGs at 25 °C with shaking (180 rpm). The cow milk and tea soups contaminated with AFB1 were used as the control. After 30 min incubation, the supernatant was extracted three times via shaking at 180 rpm with half the volume of chloroform, as described above. The residual AFB1 was assessed using HPLC. All the experiments were repeated three times.

### 5.12. Statistical Analysis

The results from the replicates are reported as the mean ± standard deviation (SD). SPSS 18.0 (SPSS, Inc., Chicago, IL, USA) was used to assess the statistical analysis via one-factor analysis of variance (ANOVA) and Tukey’s HSD test. Statistical significance was defined as *p* < 0.05 in all the experiments.

## Figures and Tables

**Table 1:** Isotherm parameters for the adsorption of AFB1 onto the WKGs.

Langmuir		Freundlich	
q_m_ (µg g^−1^)	25.773	K_f_ (µg g^−1^)	1.601
K_L_ (mL µg^−1^)	0.094	n	1.644
R^2^	0.529	R^2^	0.924

**Table 2:** Kinetic parameters for the adsorption of AFB1 onto the WKGs.

Pseudo-First-Order		Pseudo-Second-Order		Intraparticle Diffusion	
k_1_ (min^−1^)	0.067	k_2_ (g µg^−1^ min^−1^)	0.099	k_3_ (g µg^−1^ min^−1/2^)	0.392
q_e_ (µg g^−1^)	2.733	q_e_ (µg g^−1^)	6.878	b	4.456
R^2^	0.710	R^2^	0.9995	R^2^	0.663

## Data Availability

All the data that support the findings of this study are available from the corresponding author upon reasonable request.

## Supplementary Materials

The following supporting information can be downloaded at: https://www.mdpi.com/article/10.3390/toxins16020107/s1, Figure S1: Mass spectra of the removal of AFB1 mediated by WKGs; Figure S2: Mass spectra of degradation products for AFB1 by WKGs.
