# Supplementary material for: Microbial Composition of Water Kefir Grains and Their Application for the Detoxification of Aflatoxin B1
Source: Toxins (Basel). 2024 Feb 15;16(2):107. doi: 10.3390/toxins16020107 (PMC10893553; doi:10.3390/toxins16020107)
Supplement: Supplementary file 1 [file toxins-16-00107-s001.zip › toxins-2854029-supplementary.pdf]

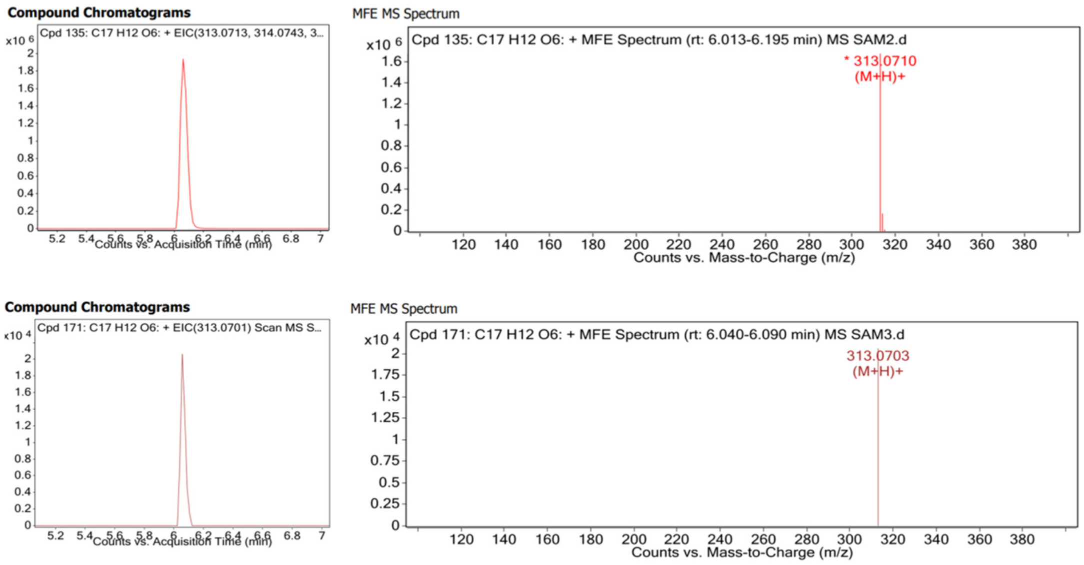

Supplementary Figure S1. Mass spectra of the removal of AFB1 mediated by WKGs. HPLC-Q-TOF-MS analyzed the content of AFB1 in brown sugar solution supplemented with 2 μg/mL AFB1(up) and co-incubation cultures of 20% of WKGs and 2 μg/mL AFB1 in brown sugar solution for 30 min (down).

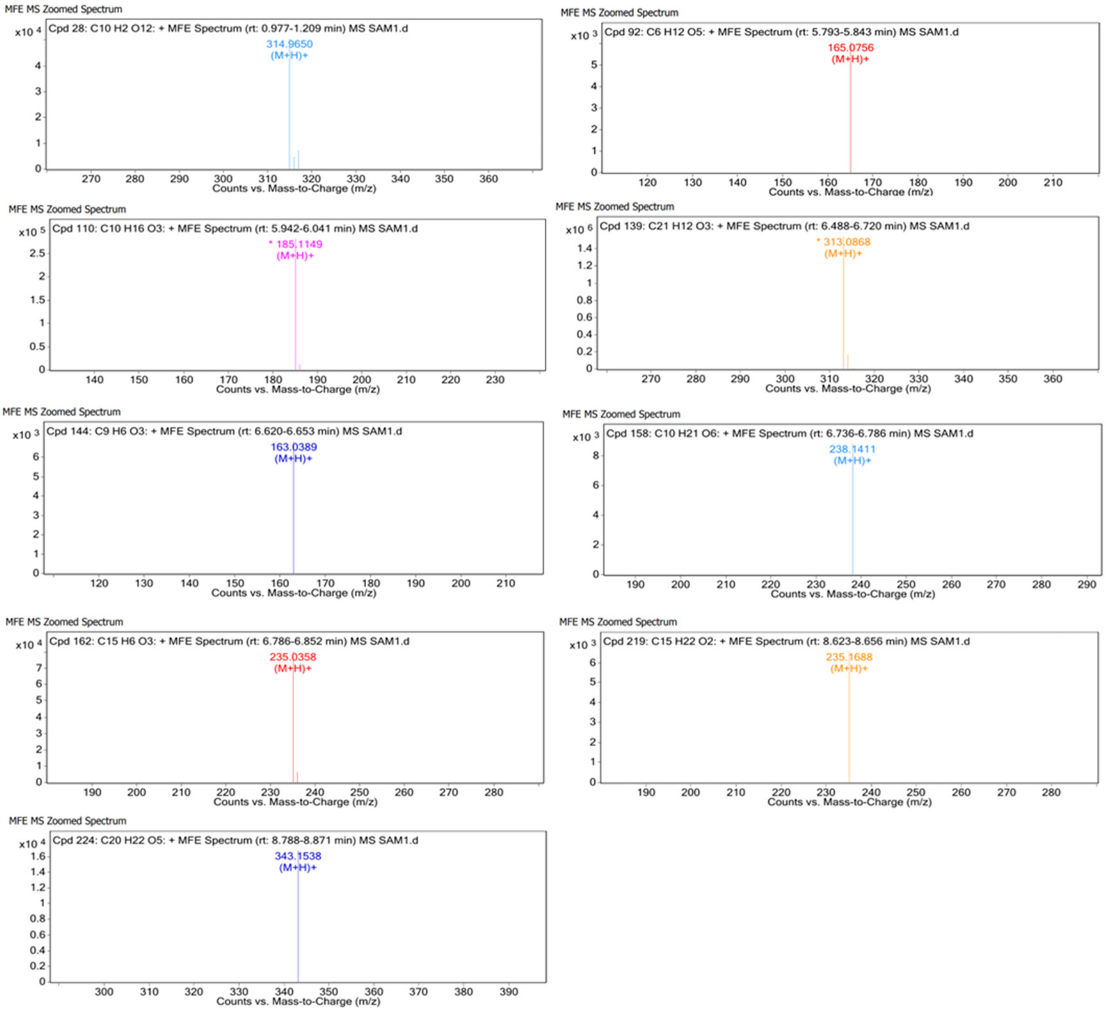

Supplementary Figure S2. Mass spectra of degradation products for AFB1 by WKGs.
